# Data of biomass and N in grass and clover roots, stubbles, and herbage and associated N_2_O and CO_2_ emissions, inclusive soil air composition, following autumn ploughing – A field study

**DOI:** 10.1016/j.dib.2022.108352

**Published:** 2022-06-04

**Authors:** Marina Azzaroli Bleken, Tatiana F. Rittl, Sandhya Karki, Shahid Nadeem

**Affiliations:** a Faculty of Environmental Sciences and Natural Resource Management, Norwegian University of Life Sciences (NMBU), Ås, Norway; b Presently Norwegian Centre for Organic Agriculture (NORSØK), Tingvoll, Norway; c Presently University or Arkansas, Biological and Agricultural Engineering, Fayetteville, Arkansas, USA

**Keywords:** Denitrification, Decomposition, Nitrous oxide, Crop residue, Low temperature, Freeze-thaw, Snow

## Abstract

This article presents the detailed data of the soil characteristics, field management, amount and N content of below- (roots +crown) and aboveground (stubble and herbage) grass mixture, red clover and red clover grass swards at the end of the 3rd production year, together with fluxes of greenhouse gas emissions (N_2_O, CO_2_, CH_4_) and soil air composition (CO_2_, N_2_O, CH_4_, N_2_ and O_2_) of a field experiment in Norway. These data supplement the findings presented in the research article “ Roots and other residues from ley with or without red clover: quality and effects on N_2_O Emission Factor in a partly frozen soil following autumn ploughing”(Bleken et al. 2022). For understanding of the effects of incorporating ley above- and belowground residues on cumulative greenhouse emissions refer to article from this research.

**Specifications Table**

**Table:** 

Subject	Agricultural Sciences, Agronomy and crop
Specific subject area	Root biomass, Ley residues and Greenhouse gas emissions from agricultural soils
Type of data	Table Figure Pictures Text file data
How data were acquired	Plant and soil material was collected in a field experiment in a silty clay loam (31% clay, 46% silt) from plots of three different forage leys (a grass mixture, a red clover-grass mixture, a red clover in pure stand) at the end of the third production year. Stubble, defined as the biomass present above ground after forage harvest, was collected from representative subplots (60 cm X 60 cm), cutting with a knife at 0–0.5 cm depth. Soil core samples (for collection of crown and roots) were taken with a root auger (ø 8cm) to 30 cm soil depth. Plant crown are defined as belowground organs where stems and roots join each other, but morphologically different from both. Roots and stubbles were carefully washed and cleaned from debris, and dried in a ventilated oven at 40 °C, then analyzed for elemental C and N (Dumas method, Leco CHN628). More quality variables are reported in [1]. Roots and crown amounts were corrected for soil minerals impurities based on the recorded total C concentration and assuming
	that the correct C content was 45% of the dry biomass. After early autumn ploughing, soil gas fluxes were estimated using manual closed chambers (inner height 19.5 cm) and two fixed aluminum frames per plot (51 × 51 × 20 cm inner size) inserted to 10–15 cm depth in plots with grass mixute and red clover grass-mixture. Chambers were equipped with a 3 mm diameter pressure equilibrium tube and with a sampling tube, assessing the middle of the chamber, ending with a three-way stopcock valve. Gas samples were collected by deploying the chambers on the frames and withdrawing 15 ml gas samples from the chamber headspace with a 20 ml polypropylene syringe at start and 45 minutes later. Before sampling, air in the chamber headspace was mixed by pulling and pushing the plunger of the syringe 3–4 times. The sample was transferred through the three-way valve to He washed, pre-evacuated 12 ml glass vials crimped with butyl rubber septa, causing an over pressure in the vials in order to avoid contamination during sample storage. There were 45 sampling events over 252 days. Sampling frequency varied and was higher when high fluxes could be expected, e.g. immediately after rain and during thawing, and also just before these events in order to identify peak fluxes. The composition of soil air was obatined using probes inserted at 8, 24, 40 cm depth. The probes had an air-permeable cup (pore Ø 100 μm) tightened to PTFE tube (inner Ø 0.97 mm) which runs through a PVC tube (outer Ø 3.3 cm) ending with a three-way stopcock valve (see [2,3] for details). They were installed into pre-augered holes, at a 60° angle to the soil surface. All gas samples were analysed using gas chromatography (GC, model 7890A, Agilant, Santa Clara, CA, USA) using a 30-m wide bore Poraplot Q (0.53 mm) column at 38 °C with back flushing and helium (He) as carrier gas. The GC was equipped with an electron capture detector (ECD), a thermal conductivity detector (TCD) and flame ionization detector (FID). The ECD was run at 375^0^ C with 17 ml min^−1^ ArCH4 (90/10 vol %) as a makeup gas. The GC was connected to an autosampler via a peristaltic pump (Gilson minipuls 3, Middleton, W1, USA), pumping approximately 2.5 ml gas into a 250 µl sampling loop maintained at 1 Atm pressure. The injection system was back-flushed by He 6.0 before each sampling to minimize memory effects.
	Soil temperature and moisture were measured using four dataloggers (EM50 datalogger, Decagon Devices, Pullman, WA, USA), conneted with temperature and mositure sensors (5TM).
Data format	Analysed Calculated
Description of data collection	Roots and aboveground residue biomass and qualities were determined in a grass mixture, a red clover in pure stand, and a red clover-grass mixture. Gas soil fluxes were recorded over 252 days in plots of the grass mixture and of the red clover-grass mixture, ploughed in at the end of the third productive year. Living grass ley and de-vegetated plots kept were used as controls. Soil air data were collected at 8, 24 and 40 cm depth from selected treatments.
Data source location	Institution: Faculty of Environmental Sciences and Natural Resource Management, NMBU: Norwegian University of Life Sciences City/Town/Region: Ås, Viken Country: Norway Latitude and longitude for collected samples/data: 59°39′47"N, 10°45′42"E
Data accessibility	Repository name: NMBU Open Research Data https://dataverse.no/dataverse/nmbu Data identification number: https://doi.org/10.18710/3JGCCO Direct URL to data: https://dataverse.no/dataset.xhtml?persistentId=doi:10.18710/3JGCCO
Related research article	M. A. Bleken, T. Rittl, S. Hansen, S. Nadeem. Roots and other residues from ley with or without red clover: quality and effects on N_2_O Emission Factor in a partly frozen soil following autumn ploughing. STOTEN [1] http://dx.doi.org/10.1016/j.scitotenv.2022.154582

**Value of the Data**

• The data combine direct measurement of quantity and quality of below- and aboveground residue from different type of forage leys, with sampling of N_2_O fluxes after autumn ploughing of the residues in the same field. These data constitute an unique contribution to the assessment of emission factors from non removable crop residues compared to emissions from harvestable residues.
• In addition to N_2_O, the dataset also includes CO_2_ emissions and CH_4_ consumption and data on soil air composition, which are important to decoupling the mechanisms behind the production of soil greenhouse gas emissions.
• Plant residue biomass data and quality are useful for plant physiologist and ecologist.
• These data are relevant for researchers working with modelling of N_2_O emission from plant residues, for agronomists and environmental scientists and for stakeholders involved in inventories of greenhouse gas emissions.
• The data can be used to parameterize, calibrate or validate models which estimate the impact of above- and belowground residues on greenhouse gas emissions. The data can also be used in meta-analysis looking for convenient methods to estimate N_2_O emission factors from plant residues.

## 1 Data Description

This article includes the descriptive data (means), analysed and calculated data on the effects of ley type on below- and aboveground plant residues and on greenhouse gas emissions and soil air composition. The data presented here include detailed information of soil characteristics and field management, and data collected in the experiment, whereas the data and statistic of the other parameters were used in the analyses reported in the related article [1]. Replicate data are available in the linked database.

**Site description.**Table 1 gives a detailed characterization of the plough layer (0–20 cm) and subsoil of an artificially drained Umbric Epistagnic Retisol soil [4] localized in Ås, Norway (59°39′47"N, 10°45′42"E). The treatments were placed in a long-term trial and utilize plots which were nearly uniquely cultivated with annual crops since 1963, before being sown with leys. The leys were ploughed at the end of the 3^rd^ productive year. In May 2015, three different leys were sawn: a grass-mixture, a clover grass mixture and a red clover in pure stand. The grass mixture proportion, botanical and cultivar names are given on Tables 2 and 3, respectively. An overview over the field history and timing of the main managements is given in Table 4.

**Table 1:** Soil characteristics in the plough layer (0–20 cm) and subsoil. Mean values and (standard deviation). Soil pore distribution and bulk density assessed in summer 2014 when the field was cropped with spring oats.

	Soil depth (cm), (n ≥ 16, sampled in 2012)		
Soil characteristics	0–20	20–35	35-50
Sand (%)^a^	22 (8.7)	32 (10)	18 (2.5)
Silt (%)^a^	46 (6.6)	39 (6.1)	50 (2.5)
Clay (%)^a^	31 (3.7)	29 (4.9)	32 (3.4)
SOC (%)	2.81 (0.35)	0.99 (0.6)	0.48 (0.2)
Total N (%)	0.25 (0.04)	0.10 (0.05)	0.07 (0.02)
C:N	11.14 (0.74)	9.5 (2.1)	6.8 (1.5)
	Plot for gas fluxes and root sampling	Limed plots, only used for root sampling	
pH_H2O_^b^, 0 -20 cm depth	5.10 (0.18), (*n* = 14)	6.05 (0.10), (*n* = 17)	
pH _CaCl2_^b^_,_ 0 -20 cm depth	4.70 (0.17), (*n* = 45)	5.77 (0.22), (*n* = 33)	
	Depth of bulk analysis samples (cm)		
	10 – 15 (*n* = 23)	25 – 30 (*n* = 24)	40 – 45 (*n* = 16)
Soil bulk density (g soil cm^−3^)^c^	1.18 (0.08)	1.51 (0.14)	1.71 (0.09)
Pore volume (% of total soil volume)^c^	54 (3.4)	44 (4.9)	37 (2.9)
0.1 kPa (Field capacity, %)^c^	36 (2.4)	35 (3.8)	29 (3.5)
Water filled volume,1 kPa (%)^c^^,^^d^	31 (2.0)	33 (4.0)	27 (3.6)
Water filled volume, 15 kPa (Permanent wilting point, %)	11 (1.4)	18 (4.8)	17 (3.8)
Air permeability (μm^2^)	42 (23)	9 (7)	3.6 (1.7)

a) Data are from an unpublished study on the same field (Bleken, Børresen and Krogstad), susceptible to minor changes due to recalibration of the methods used.

b) Sampled spring 2019. Soil dried at ambient temperature.

c) Assessed in a previous cereal crop (2014) during the growing season. In the plough layer the initial porosity is likely underestimated, and so the water content at field capacity is overestimated compared to the conditions shortly after ploughing. This thus not affect the wilting point, which depends on the soil texture and SOM content.

d) Lower limit for easily available water

**Table 2:** Grass mixture seeding proportion (%).

Name	Short name	% Timothy	% Perennial Ryegrass	% Meadow fescue	% Tall fescue	% Red clover
Grass mixture	G	20	40	20	20	-
Red Clover	R	-	-	-	-	100
Red clover - grass mixture	CG	16	32	16	16	20

**Table 3:** Botanical and cultivar names of the grasses and red clover used in this study.

Species	Botanical name	Cultivar
Timothy	*Phleum pretense* L.	Grindstad
Prennial ryegrass	*Lollium perenne L.*	Figgio
Meadow fescue	*Schedonorus pratensis (Huds.)* P.	Fure
Tall fescue	*Schedonorus arundinaceus* (Schreb.) Dumort	Swaj
Red clover	*Trifolium Pratense* L.	Lea

**Table 4:** Timeline of field management and main operations.

Year	Date	Main field operations
1953-2013		Spring cereal rotation (barley, wheat, oats) or spring cereals rotation with other annual crops (spring cereals every second year, potatoes, fodder beat, and later mainly field mustard
2014	September	Liming: 23 t ha^−1^ dolomite on randomized plots used in the root study. The dolomite was distributed in two doses, and the soil was ploughed to 20 cm with a moldboard plough after the first application, than harrowed to 10 cm depth after the second application. Gas sampling occurred only on low pH plots (pH 5.10)
2014	Autumn	Ploughing; 18-20 cm soil depth after spring oats in 2014
2015	12.05.	Harrowing; 3-5 cm soil depth
2015	18.05.	Barley sown; 150 kg seed ha^−1^ of spring barley sown as cover crop; Fertilization, 100 kg N ha^−1^.
	29.05.-04.06	Grass mixture, clover-grass mixture and red clover pure stand sown. Ley type x liming treatments were completely randomized and replicated 4 times; 30 kg seed ha^−1^.
2016		Fertilized in early spring, after 1st and after 2nd harvest, totally ∼140 kg N ha^−1^y^−1^ on CG and ∼270 kg N ha^−1^y^−1^ on G, no fertilizer on R. Harvested three times per year, average annual yields were 9.95, 12.7, and 13.3 Mg DM ha−1y−1 for R, G, and CG, respectively.
2017	May to September	
2018		Area fallowed in 2018 (F) was with low dose N (∼140 kg N ha^−1^y^−1)^ I in 2016 and 2017, and none in 2018
2018	07.05.	Fallow established on previous low-N dose G plots: vegetation including stubble and turf with part of the roots removed; Irrigation 30 mm
	01.06	1^st^ harvest
	06.06.	Fertilization II; 44 kg N ha^−1^ on CG and 98 kg on G; irrigation 20 mm
	07.06.	Irrigation 20 mm
	11.07	2^nd^ harvest
	12-13.07.	Fertilization III; 40 kg N ha^−1^on CG and 60 kg N ha^−1^on G
	05.08.	Irrigation 15-25 mm
	27.08.	Irrigation 30-40 mm, mainly to facilitate sampling of roots
	04.09	3^rd^ harvest
	06-08.09	Sampling of roots and stubble
	17.09.	Ploughing
	18.09.	Harrowing Frames installed
	19.09	First flux sampling event
2019	29.05	Last gas sampling event

**Fig. 1:**
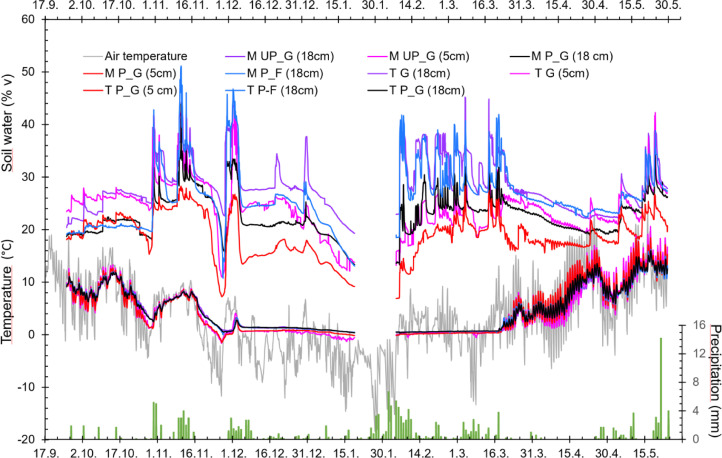
Soil moisture (% by volume, upper lines, M), air temperature (°C) and soil temperature (°C, T), and precipitation (mm, right vertical axis). Temperature and soil moisture are hourly averages. M: moisture and T: temperature of P_G: ploughed grass, P_F: ploughed fallow, and of grass ley (M UP_G and T G).

Soil moisture and temperature, air temperature and precipitation are shown in Fig. 1.

**Plant organs biomass and N amount**. The sums of belowground biomass (crown and roots) and N per ha, their standard deviation and share in % of the belowground biomass are given on Table 5. Roots biomass were sampled after the 3^rd^ harvest of the swards in 2018, between 6 and 8 September 2018, whashed and crowns were separated from roots (Picture 1**)**. The treatments and the total amount of dry biomass and N in each plant part is presented on Table 6.

**Table 5:** Total amount of biomass (DM) and total nitrogen (Tot N) in belowground residues. Averages of low and high pH plots as there was not statically significant difference between them. *n* = 8.

		Biomass						Total N					
		Grass		Red clover		Clover-grass		Grass		Red clover		Clover-grass	
Depth (cm)	Plant part	Mean	SD	Mean	SD	Mean	SD	Mean	SD	Mean	SD	Mean	SD
		(Mg DM ha^−1^)						(kg N ha^−1^)					
0-15	Crown	1.83	1.27	1.59	1.25	1.6	1.31	30.14	22.24	34.58	27.25	26.69	22.91
	Root	7.39	2.11	7.24	2.22	6.78	2.49	135.13	41.49	190.38	65.96	156.47	59.71
15-23	Root	0.98	0.29	0.86	0.33	1.31	0.31	21.36	6.13	24.1	8.89	32.37	7.88
23-30	Root	0.34	0.19	0.3	0.18	0.49	0.1	6.74	4.42	8.32	5.02	12.01	2.33
0-30 cm	Sum	10.54	2.84	9.99	2.97	10.17	3.21	193.37	54.64	257.37	85.73	227.53	70.96

Share of total (%)													

0-15	Crown	17		16		16		16		13		12	
	Root	70		72		67		70		74		69	
15-23	Root	9		9		13		11		9		14	
23-30	Root	3		3		5		3		3		5	
													
0-15	Crown +root	0.87		0.88		0.82		0.85		0.87		0.80

**Table 6:** Total biomass (DM) and nitrogen in the plant residues incorporated after ploughing (P) of a grass (G) or red clover-grass ley (CG) at the end of the 3rd production year, without or with the herbage of the last harvest kept as green manure (M).

Treatment	Ley mixture	Ploughed	Residue amounts	Herbage	Stubble	Roots	Total
P_CG	Red clover-grass	Yes	t DM ha^−1^	0	5.49	10.2	15.7
			kg N ha^−1^	0	99	228	327
P_CG_M	Red clover-grass	Yes	t DM ha^−1^	3.45	5.49	10.2	19.1
			kg N ha^−1^	97	99	228	424
P_G	Grass	Yes	t DM ha^−1^	0	4.33	10.5	14.9
			kg N ha^−1^	0	60	193	253
P_G_M	Grass	No	t DM ha^−1^	3.6	4.33	10.5	18.5
			kg N ha^−1^	66	60	193	319
P_fallow	Grass removed spring 2018	Yes		No residue applied, Grass and turf removed spring 2018			
Ley	Grass	NO		No residue applied -			

**Soil greenhouse gas emissions.**Table 7 presents the mean cumulative N_2_O emission (g N ha^−1^) and CO_2_ emission (kg C ha^−1^) calculated per period and total over 252 days. Means of 4 replicates (± standard deviations) were grouped for different periods: Fall from 19/09/2018 to 28/10/2018; Freeze-Thaw from 29/10/2018 to 5/12/2018; Snow from 6/12/2018 to 11/02/2019; Melting from 12/02/2019 to 24/03/2019; Spring from 25/03/2019 to 29/05/2019. Table 8 shows the cumulative CH_4_ emissionsfor the whole experimental period.

**Table 7:** Cumulative N_2_O emissions (g N ha^−1^) and CO_2_ emissions (kg C ha^−1^) per period and totally during 252 days after ploughing (P) of a grass (G) or red clover-grass ley (CG) at the end of the 3rd production year, with or without the herbage of the last harvest kept as green manure (M). Means of 4 replicates (± standard deviations). Subsequent periods: Fall from 19/09/2018 to 28/10/2018; Freeze-Thaw to 5/12/2018; Snow: to 11/02/2019; Melting: to 24/03/2019; Spring: to 29/05/2019. One or more letters in common indicates no statistically significant difference (LSD-test, *p* > 0.05).

	Fall			Freeze-Thaw			Snow			Melting			Spring			Total			Total without melting		
Treatments	Mean		SD	Mean		SD	Mean		SD	Mean		SD	Mean		SD	Mean		SD	Mean		SD
	N_2_O emission (g N ha⁻¹)																				

P_CG	174	b	90	244	b	338	85	a	82	385	a	283	272	a	93	1160	ab	489	775	ab	420
P_CG_M	423	a	261	374	a	211	91	a	66	751	a	1075	352	a	201	1991	a	1643	1240	a	688
P_CG_M^a^										406		394				1646		824			
P_G	75	d	19	98	bcd	40	25	bc	31	485	a	482	288	a	137	970	b	471	486	bc	180
P_G_M	138	bc	51	118	bc	76	105	a	77	286	a	170	326	a	145	970	ab	272	687	ab	240
P_Fallow	91	cd	47	68	cd	31	9	c	13	94	b	97	112	b	62	370	c	129	280	c	85
Ley	81	d	29	49	d	22	38	ba	18	19	c	13	138	b	127	320	c	177	306	c	174

	CO_2_ emission (kg C ha⁻¹)																				

P_CG	539	b	126	173	c	62	62	ab	43.8	79	a	47.5	400	bc	81	1250	c	169	1174		182
P_CG_M	1382	a	914	257	b	49	74	a	25.1	132	a	167.3	471	b	225	2320	b	1165	2184		1056
P_CG_M^a^										81		25				2264		1051			
P_G	583	b	159	224	bc	45	58	ab	31.9	133	a	129.9	381	bc	108	1380	c	304	1245		252
P_G_M	835	a	118	240	b	62	114	a	67.7	78	a	54.1	408	bc	50	1680	cb	159	1598		140
P_Fallow	262	c	59	121	d	49	35	b	27.1	23	b	28.4	303	c	70	740	d	148	721		142
Ley	842	ab	220	427	a	154	104	a	38.0	67	a	19.0	2126	a	281	3560	a	541	3498		533

a : if the single highest emission value observed during melting is considered an outlier and replaced by the average of the other 7 chambers in the same treatment before integration. The results of LSD means comparison during Melting do not change, while for the Total emissions, treatments with ploughed leys are similar to each other and all of them different from each of P_Fallow and Ley, which are similar to each other.

**Table 8:** Cumulative soil CH_4_ emission (mg C m^−2^) during 252-day field experiment after ploughing (P) of a grass (G) or red clover-grass ley (CG) at the end of the 3rd production year, without or with the herbage of the last harvest kept as green manure (M). Means of 4 replicates (± standard deviations). One or more letters in common indicates no statistically significant difference (LSD-test, *p* > 0.05).

Treatments	CH_4_-C (g C ha^−1^)
P_CG	-192 (18.5) a
P_CG_M	-278 (15.4) ab
P_G	-130 (9.4) ab
P_G_M	-170 (16.4) ab
P_Fallow	-177 (8.7) ab
Ley	-120 (12.3) b

**Fig. 2:**
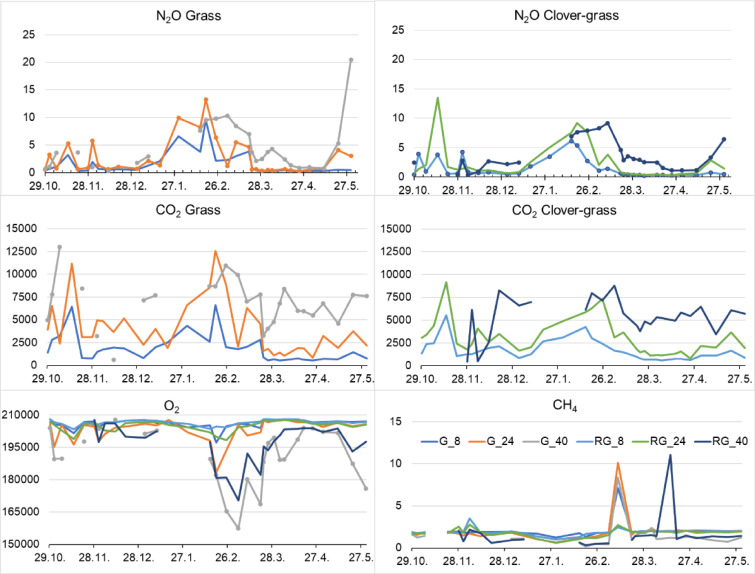
Partial pressure of N_2_O, CO_2_, O_2_ and CH_4_ in soil air sampled at 8, 24 and 40 cm depth under ploughed grass ley (P_G, in the legend called G) and ploughed red clover-grass ley with the last herbage harvest kept before ploughing (P_CG_M, in the legend called RG). Averages of four replicates. All gas partial pressure in ppm.

**Soil air content** of N_2_O, CO_2_, O_2_ and CH_4_, in soil air sampled at 8, 24 and 40 cm depth is given as partial pressure (ppm) in Fig. 2.

**Soil Mineral N.** Means of 4 replicates (± standard deviations) of the amount of NH_4_ (kg ha^−1^) and NO_3_ (kg ha^−1^) and total soil mineral N (kg ha^−1^, 0-20 cm depth) measured five times from Spring 2018 (03/09/2018) to Spring 2019 (29/05/2019) in different treatments are given in Table 9.

**Table 9:** Soil Mineral N amount during 252-day field experiment after ploughing (P) of a grass (G) or red clover-grass ley (CG) at the end of the 3rd production year, without or with the herbage of the last harvest kept as green manure (M) SD: standard deviation, n=4, except before ploughing when *n* = 8.

			Soil Mineral N (kg ha^−1^)				
Day	Period	Treatment	NH₄⁺	SD	NO₃⁻	SD	TOTAL
03.09.2018	Before ploughing	P_CG	7.71	4.98	2.70	1.59	10.41
		P_CG_M					
		P_G	3.40	0.52	0.76	0.26	4.16
		P_G_M					
		P_Fallow	5.59	1.67	1.10	0.94	6.70
		Ley	3.40	0.52	0.76	0.26	4.16
							
03.10.2018	Fall	P_CG	15.34	10.88	10.19	3.45	25.53
		P_CG_M	9.75	3.34	12.36	5.06	22.11
		P_G	6.18	1.62	3.49	1.25	9.68
		P_G_M	8.75	4.45	4.79	1.40	13.55
		P_Fallow	5.32	1.54	6.64	3.38	11.96
		Ley	4.61	1.70	3.06	1.63	7.67
							
07.11.2018	Freez-Thaw	P_CG	3.52	0.95	8.20	0.96	11.73
		P_CG_M	2.87	0.68	8.30	2.42	11.17
		P_G	3.06	0.91	2.69	0.99	5.76
		P_G_M	2.44	0.78	4.16	1.10	6.60
		P_Fallow	2.12	0.29	5.69	1.71	7.81
		Ley	2.24	0.76	1.57	0.79	3.81
							
29.03.2019	Melting*	P_CG	2.57	0.95	4.17	1.22	6.74
		P_CG_M	1.49	0.92	6.07	4.32	7.56
		P_G	2.71	0.63	3.40	0.46	6.11
		P_G_M	1.70	0.77	4.34	1.05	6.04
		P_Fallow	2.12	0.93	4.16	0.30	6.28
		Ley	3.89	1.84	3.17	1.49	7.07
							
29.05.2019	Spring	P_CG	3.50	1.27	16.03	7.92	19.53
		P_CG_M	4.13	2.02	16.86	5.72	21.00
		P_G	2.48	0.71	8.57	5.38	11.05
		P_G_M	3.58	2.35	14.96	4.04	18.53
		P_Fallow	3.15	1.05	9.70	4.67	12.85
		Ley	4.14	1.01	5.83	6.48	9.98

⁎ Sampled 4 days after melting period finished.

The **pictures** show details of field experiment, plant organs and method.

Picture 1. Details of the root samples and morphology.

**Picture 1:**
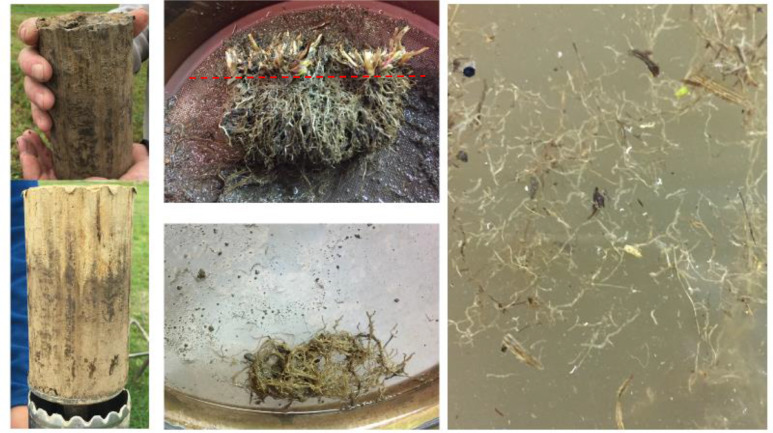
Left: Root core samples upper and lower layer (turned upside-down) collected with a root auger (inner Ø 8 cm). Middle: detailed of the root collected with crown (above the red line, originally below ground surface) in the field. Right: roots dispersed in water for removal of other debris.

Picture 2. Detail of the frame and chamber used for gas sampling.

**Picture 2:**
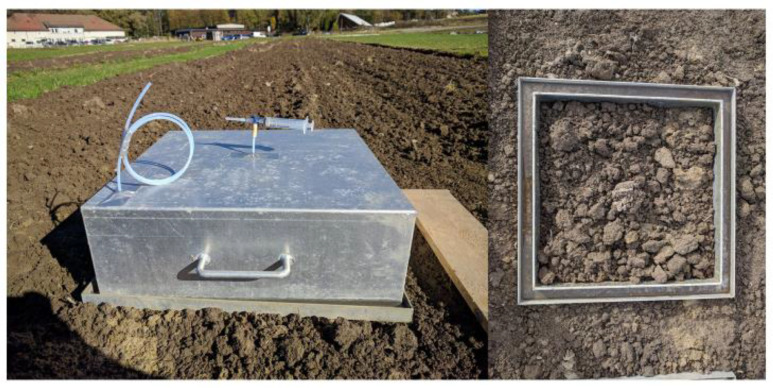
Left: chamber used in the field experiment for gas sampling. Right: frame place in the field experiment.

Picture 3. Mulching of the herbage in the experimental plot just before ploughing

**Picture 3:**
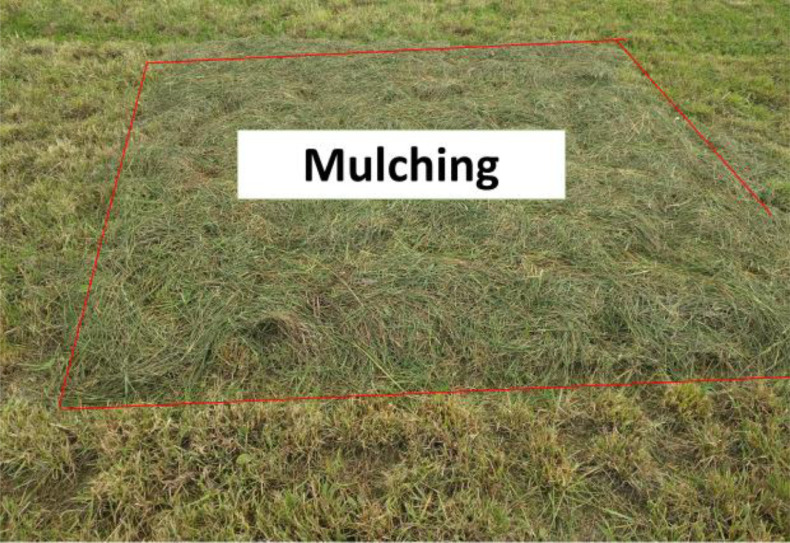
Detail of the plots with green manure.

Picture 4. Detail of the installation of the probes for soil air sampling.

**Picture 4:**
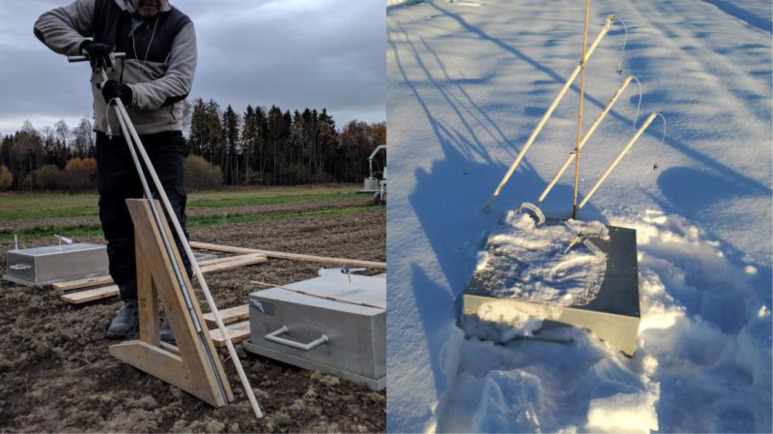
Left: Inserting the tube probes to sample soil air. Right: probes and gas chamber during winter.

Picture 5. Soil structure and weather condtions at during the autumn 2018

**Picture 5:**
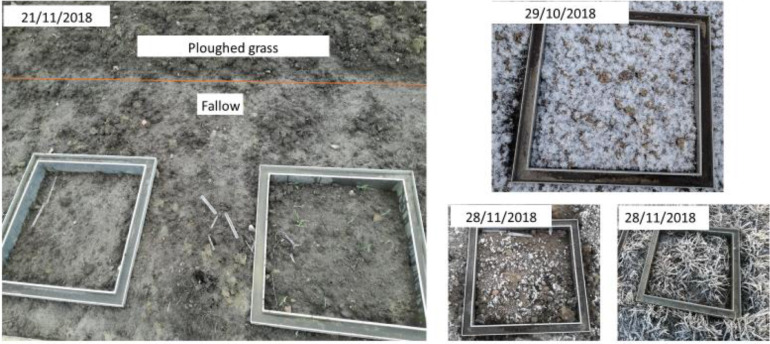
Left: Difference in soil structure in the ploughed soil and fallow, picture taken during a thaw period in between to freezing periods. Right; two Freezing events during autumn in different treatments.

The **dataset** named “Replication data for: Roots and other residues from leys with or without red clover; quality and effects on N_2_O Emission Factor after ploughing” in the NMBU Open Research Data repository, contains the following files:

• 00_README file.txt: contains the detailed description of the dataset uploaded, i.e. treatments, units, abbreviations, date, etc.
• 01_Gas_Data_field-.txt: Flux data of N_2_O, CO_2_ and CH_4_ from autumn 2018 to spring 2019. Single frames values, 8 frames per treatment distributed pairwise on four plots. 02_Gas_Data_Cumulated.txt: Cumulated emission by linear integration of N_2_O, CO_2_ and CH_4_ by period from autumn 2018 to spring 2019 - 8 frames per treatment distributed pairwise on four plots.
• 02_Gas_Data_Cumulated.txt: cumulative emissions of N_2_O, CO_2_ and CH_4_ by period and totally from autumn 2018 to spring 2019 - 8 frames per treatment distributed pairwise on four plots.
• 03_Soil_Moisture_Temperature.txt: Soil moisture (volumetric moisture, %) and temperature of soil (°C) on selected treatments at two depths (cm). Each value is the average of four probes, distributed on four plots. Occasionally a probe did not function, this is not shown in the data.
• 04_Soil_Mineral_Nitrogen.txt: Amount of ammonium-N (NH_4__N) and nitrate-N (NO_3__N) in the soil layer (0-20 cm depth) at selected dates, in kg N per ha, soil bulk density = 1.15. Replicate values, n=4.
• 05_Root_Crown_amounts.txt: Biomass dry weight and amount of total nitrogen (N), nitrate and ammonium, water soluble carbon (C) in roots and crown.
• 06_Quality_roots_crown_stubble_herbage.txt: Concentration of nitrogen, nitrate, ammonium, water soluble carbon, van Soest fractions (NDF: neutral detergent fiber, ADF: acid detergent fiber, ADL: acid detergent lignin) and derived biochemical compounds composition (ND-soluble, Hemicellulose and cellulose) in dry biomass of roots, crown, stubble and herbage used in the field experiment.
• 07_Experimental_design.pdf: schematic plan of main plots and treatments used for root and gas sampling
• 08_Field_position.pdf: position of the field seen aerial photo and placed on map of the NMBU campus
• 09_Pictures.pdf: Photos of field details during different periods

## 2 Experimental Design, Materials and Methods

### 2.1 Study Site

A field experiment was conducted from September 2018 to May 2019 in Ås, Norway (59°39′47"N, 10°45′42"E), on an Umbric Epistagnic Retisol soil [4], where the normal (1971–2000) annual mean temperature is 5.7°C and precipitation is 795 mm (NMBU weather station, Ås,59°39′37.8"N, 10°46′54.5"E). The experiment was established on the previously long-term (1953-2013) crop rotational field trial at aresearch farm of the Norwegian University of Life Sciences (NMBU) Ås [5]. Plots with similar cultivation history and nearly always annual crops since 1963 were selected for this study. The soil is artificially drained at about 1 m depth.. The soil is a silty clay loam (31% clay, 46% silt) in the upper layer plough layer (o-20 cm), with a naturally compacted subsoil below the plough layer. Soil organic carbon (SOC) in the upper soil layer is ∼2.8 % and decreases rapidly below ploughing depth in connection with a jump in soil bulk density and a drop in the larger pores. The soil pH was low (pH_CaCl2_ 4.7 in 10 mM CaCl_2_, pH_H2O_ 5.1, in spring 2019) due to absence of liming since 1970. In autumn (late September- October) 2014, part of the plots were limed with 23 t ha^−1^ dolomite distributed into two doses, one before ploughing to 20 cm depth and the other before harrowing to 10 cm depth, which raised the soil pH_CaCl2_ to 5.8 or pH_H2O_ to 6.05 (limed) in spring 2019 (Table 1). Weather data was collected from the nearby NMBU weather station in Ås as mentioned above.

### 2.2 Leys Management

Three different leys were sawn end of May 2015, only grasses (G), clover-grass mixture (CG) and red clover in pure stand (R) (Tables 2 and 3; dataset file 07). Each ley treatment was replicated 4 times and fully randomized. On both G and CG, each plot was divided longitudinally into two subplot (2.7 m X 10 m), one receiving half (140 kg N ha y^−1^) and one receiving normal (270 kg N ha y^−1^) N fertilization rate, distributed in early spring (40%), after the 1st harvest (30%) and after the 2nd harvest (30%). Red clover in pure stand (R) did not receive any fertilizer. From 2016 to 2018 the herbage was harvested three times per year (Table 4), at the plant stages recommended for high quality silage, with a Haldrup F-55 grass harvester (J. Haldrup a/s, Denmark). At the beginning of the growing season in 2018 (07 May), a de-vegetated control (fallow) treatment was also established on a subplot (2.5 m x 2.7 m) in each low nitrogen G plots by breaking the sod with a handhold cultivator then removing manually the turf and regularly removing any weed. No fertilizer was applied to these fallow plots in 2018. At the end of 3rd production years (2018), after the 3^rd^ harvest on September 4, all plots were ploughed with a moldboard plough on September 17 to 20 cm depth. However, a 3 m wide area of each plot was left non-ploughed as living ley.

### 2.3 Roots and Stubble Sampling, and N Analysis of Plant Substrates

On 6–8 September 2018, roots samples were taken from grass (G, full dose N), red clover-grass (CG, half dose N) and red clover (R, no N fertilizer) plots, both in non-limed and limed plots (dataset file 07). Two root core samples were collected with a root auger (inner Ø 8 cm) from each of 8 replicate plots (4 on low pH and 4 on limed plots), thus 16 cores per ley type distributed pairwise on the row and in between the plants’ rows. Cores were taken from 0–15 cm, and 15–30 cm soil depth; the latter was divided in 15-23 cm and 23-30 cm soil depth and stored at 1°C until washing in cold water. Visible particles of parts of decomposed dead plant residues were removed and all water used was sieved through a 35 μm sieve to ensure that no root piece was lost. The crown, which here is defined as belowground plant tissues connecting shoots and roots, was kept separate from the roots. Thus, belowground residues include both roots and crowns. Stubbles, here defined as the amount of biomass present aboveground after harvest, were collected from representative areas (60 cm x 60 cm) on 10 September 2018, and washed. Before ploughing, herbage was harvested at 5-7 cm cutting height. All the plant materials, including herbage mentioned later, were dried under strong ventilation at 40 °C to constant weight. Finely ground samples of roots, crown, stubble and herbage were analyzed for total C and N content (Dumas method, Leco CHN62), water soluble organic C, and KCl extracted ammonium and nitrate at our Soil Science laboratory, NMBU. Neutral detergent fiber (NDF), acid detergent fiber (ADF) and acid detergent lignin (ADL) were determined following the Van Soest components method [6,7] at Artemis Laboratories (Janze, France). Based on those analysis, the following biochemical components were calculated in % of ash free dry matter (DM): ND-soluble (100 - NDF %), hemicellulose (NDF % – ADF %), cellulose (ADF % – ADL %) and lignin (= ADL). Root biomass was corrected for soil mineral impurities which remained attach to the root surface using the observed C concentration of the root sample (which was ≤ 45%) and assuming that the correct C concentration in the dry matter was 45%, as observed in a few root samples, and close to that of herbage and stubble (Tables 5 and 6; dataset files 05 and 06).

### 2.4 Gas Flux Treatments

Only the treatments grass mixture (G, full dose N) and red clover-grass (CG, half dose N) were used for gas flux measurements (dataset file 07). Before ploughing, fresh herbage, taken from adjacent field and similar to that removed at the third harvest, was applied on 2.5 m × 2.75 m subplots (3400 kg DM ha^−1^ on CG and 3600 kg DM ha^−1^ on G). This herbage amount resembles a situation when the last harvest is retained as green manure, or regrowth in excess of farm's demand is left unutilized. By that, 4 treatments were established: the G and CG plots harvested and ploughed (**P_G** and **P_CG**), and pairwise adjacent to them those with retained herbage (green manure) and ploughed (**P_G_M and P_CG_M**). To these, two reference treatments were added: the de-vegetated and ploughed (**P_Fallow**, sometimes also shortened to **P_F**), and the part of the G plots kept with undisturbed living ley (**Ley**). In total there were 24 plots with six treatments **(P_G, P_G_M, Ley, P_CG, P_CG_M and P_CG_M)** in 4 replicates per treatment.

### 2.5 Measurements of N_2_O, CO_2_ and CH_4_ fluxes

Gas samples were collected on 45 dates over a period of 252 days, from 19 September 2018 to 29 May 2019, using the closed chamber method. After ploughing on 17 September 2018, the edges of the ridges were smoothened by one pass with a light spring-tooth harrow mounted on a light tractor, to avoid ridges being higher than the metal frames described below. In each selected plot, immediately after harrowing, two micro plots per subplot were established by inserting aluminum frames (51 × 51 × 20 cm inner size) to 10-15 cm depth into the soil, in total 48 permanent frames (6 treatment × 4 replicates × 2 frames/plot), carefully placed on areas undisturbed by the wheel track at harrowing and at minimum 50 cm inside the border of the treatment. The frames upper edge ended with a groove (3 × 3 cm) which remained just above the soil ridges, which was filled with water before deploying the static chamber on it to ensure an air-tight connection [2]. The height from the base of groove to the soil surface was measured on a 28 points grid to calculate the exact volume of frame. The chambers (56 × 56 × 19.5 cm) were equipped with a 3 mm diameter pressure equilibrium tube and with a sampling tube (assessing the middle of the chamber) ending with a three-way stopcock valve. Gas samples were collected by deploying the chambers on the frames, 24 chambers at the time, and withdrawing 15-ml gas samples from the chamber headspace with a 20 ml polypropylene syringe at start and 45 min later. First sample was taken right after the deployment of chamber, while the 2nd sample was taken at 45 min, thus making a total of 96 samples on each single measurement day. Before sampling, air in the chamber headspace was mixed by pulling and pushing the plunger of the syringe 3-4 times. The sample was transferred through the three-way valve to He washed, pre-evacuated 12 ml glass vials crimped with butyl rubber septa resulting an over pressure in the vials to avoid contamination during sample storage. Gas sampling was completed in two rounds in reverse plot order, one for each of two frames on a plot.

Gas samples were analyzed at our soil biology laboratory (NMBU) using a gas chromatograph (GC, model 7890A, Agilant, Santa Clara, CA, USA) using a 30-m wide bore Poraplot Q (0.53 mm) column at 38^°^C with back flushing and helium (He) as carrier gas. The GC was equipped with three different detectors, an electron capture detector (ECD, for low concentration of N_2_O), a thermal conductivity detector (TCD, for CO_2_, N_2_O, CH_4_, O_2_, N_2_) and flame ionization detector (FID, for CH_4_). The ECD was run at 375^°^C with 17 ml min^−1^ ArCH4 (90/10 vol %) as a makeup gas. The GC was connected to an autosampler via a peristaltic pump (Gilson minipuls 3, Middleton, W1, USA), pumping approximately 2.5 ml gas into a 250 µl sampling loop maintained at 1 Atm pressure. The injection system was back-flushed by He 6.0 before each sampling to minimize memory effects. From the concentration difference between time 0 and 45 min and by considering the headspace volume of chambers and frames, fluxes of N_2_O, CO_2_ and CH_4_ were calculated on a per area basis as:

F = d/dt × V_c_ /A × M_n_ / V_m_ × 60

Where F is the emission flux (µg m^−2^ h^−1^), d/dt is the rate of change in gas concentration (ppmv min^−1^) in the chamber headspace, Vc is the volume of the chamber mounted on the frame (L), A is the area covered by the frame (m^2^), Mn is the molecular mass (g mol^−1^) of gas (N in N_2_O and C in CO_2_ and CH_4_), and Vm is the molecular volume of gas at chamber temperature (L mol^−1^) (Tables 7 and 8; dataset files 01 and 02).

Out of a total 2160 fluxes measures, 15 were missing due to mistake or removed due to suspect gas leakage as suggested by negative CO_2_ emission values. Eight of the suspected leakages were on November 28^th^, under windy and icing forhold, during the second measurement round when no water was added in the grooves to avoid ice formation (freezing). After that date, when the snowpack was high, and the frames under it were not visible, chambers were placed directly on the snow. Out of 2145 valid flux measurements, 223 were negative. The lowest was -5.74 (µg m^−2^ h^−1^) and their average -1.18 μg m^−2^ h^−1^. Occasionally they were accompanied by similarly small negative CO_2_ fluxes. These values were included in the calculation of cumulative fluxes, assuming that they were fluctuations around zero emissions, and at times also real but small N_2_O consumption. Cumulative gas fluxes were calculated by linear interpolation between the two sampling dates.

### 2.6 Composition of Soil Air and Accumulation of N_2_O in the soil

Probes for sampling soil air were inserted at 8, 24 and 40 cm depth in each P_G and P_CG_M plot. The probes had an air-permeable cup (pore Ø 100 μm) tightened to PTFE tube (inner Ø 0.97 mm) which runs through a PVC tube (outer Ø 3.3 cm) ending with a three-way stopcock valve (see [2,3] for details). They were installed into pre-augered holes, at a 60° angle to the soil surface in order to minimize preferential water flow along the tubes. At each sampling, 10–15 mL gas sample were withdrawn using a 20 ml plastic syringe and transferred into 12 mL pre-evacuated glass vials. Occasionally it was not possible to extract an air sample due to high water content which enter the probe, or because there was no sufficient air flux. Soil air samples were analyzed as described above for CO_2_, N_2_O, CH_4_, N_2_ and O_2_ gas concentrations. N_2_O concentrations (ppm) were converted to N mass assuming equilibrium between gaseous and dissolved N_2_O at the given soil temperature. The soil volume was divided into three layers, 0-20 cm; 20–30 cm and 30–45 cm soil depth, centred around the probes (inserted at different depths), and the soil air sampled was assumed to be representative for the whole layer.

### 2.7 Soil Mineral N and pH

Soil samples for determination of NH_4_^+^ and NO_3_^−^ content were taken from the plough layer (0-20 cm) before ploughing and at four dates during the experimental period (Table 9, dataset file 04). Eight soil cores per sample were taken from each G or CG plot before ploughing, thereafter four cores per sample, one core between the two frames and the other three randomly around the frames in the plot. Sieved samples (< 2mm) were partitioned into 30-35 g samples and frozen (-20°C) on the day of collection. Before extraction the frozen samples were placed overnight at +3°C, then each sample was added 120 ml 1M KCl, shaken for 1 hour at 125 rpm at room temperature. Supernatants were allowed to settle down for 10 minutes and 1ml of supernatant was transferred to 1.5ml eppendorf tube and centrifuged at 10,000 g for 15 minutes at 4°C. NO_3_^−^ and NH_4_^+^ concentration in the supernatant were determined colorimetrically by using a plate reader (Infinite F50, TECAN Austria GmbH), using the Berthelot reaction [8] for NH_4_^+^, and the Griess reaction [9] for NO_3_^−^. The presence of NO_2_^−^ was checked before reduction of NO_3_− to NO_2_^−^ through vanadium chloride [10].

In spring 2019 composite soil samples were taken from all plots at 0-20 cm depth, air dried, and analyzed for pH in 10 mM CaCl_2_, and a subset was analyzed for pH in water (Table 1).

## 3 Soil Temperature and Moisture

Four dataloggers (EM50 datalogger, Decagon Devices, Pullman, WA, USA), one per each replicate, were installed on 25 September 2018. Each logger was connected with temperature and moisture sensors (5TM), two placed at 5 cm depth (one in P_G and one in G_Ley) and three installed at 45° angle from 18 cm depth (under P_G, G_Ley and P_Fallow), (Dataset file 03). Precipitation and air temperature data were retrieved from a nearby weather station at NMBU (59°39’37.8”N, 10°46’54.5”E, 93. a.s.l.)

## Ethics statements

The authors declare that there are no ethical issues with the data presented and the methods used

## CRediT authorship contribution statement

**Marina Azzaroli Bleken:** Conceptualization, Investigation, Methodology, Formal analysis, Writing – review & editing, Visualization, Project administration, Funding acquisition. **Tatiana F. Rittl:** Data curation, Writing – original draft, Visualization. **Sandhya Karki:** Investigation, Data curation. **Shahid Nadeem:** Investigation, Data curation, Writing – review & editing.

## Declaration of Competing Interest

The authors declare that they have no known competing financial interests or personal relationships which have or could be perceived to have influenced the work reported in this article.

## Data Availability

Replicate data on: Roots and other residues from leys with or without red clover; quality and effects on N2O Emission Factor after ploughing (Original data) (DataverseNO).
